# A Randomized, Double-Blind, Graded Dose-Response Study of Norepinephrine Administration for Prevention of Post-Spinal Hypotension during Elective Cesarean Delivery

**DOI:** 10.3390/jcm12206437

**Published:** 2023-10-10

**Authors:** Volkan Baytaş, Süheyla Karadağ Erkoç, Menekşe Özçelik, Derya Gökmen, Ahmet Onat Bermede, Özlem Selvi Can, Asuman Uysalel

**Affiliations:** 1 Department of Anaesthesiology and ICM, School of Medicine, Ankara University, 06230 Ankara, Türkiye; volkanbaytas@yahoo.com (V.B.); obermede@ankara.edu.tr (A.O.B.);; 2 Department of Biostatistics, School of Medicine, Ankara University, 06290 Ankara, Türkiye; oztuna@ankara.edu.tr

**Keywords:** norepinephrine, post-spinal hypotension, cesarean delivery, perioperative outcome

## Abstract

Norepinephrine has been recently introduced for prophylaxis against post-spinal hypotension during cesarean delivery; however, no data are available regarding its optimum dosing scheme. The primary objective of this study is to compare three different infusion and bolus dose combinations of norepinephrine for prophylaxis against post-spinal hypotension during cesarean delivery. This randomized, double-blind study was performed between February 2021 and May 2022. The study protocol was registered at Clinicaltrials.gov with the identification number NCT04701190. A total of 192 parturients were enrolled into this study. Patients were assigned to three groups—Zero-Bolus High-Infusion (Group ZBHI, 0 µg/0.1 µg kg^−1^ min^−1^, *n* = 61), Moderate-Bolus Moderate-Infusion (Group MBMI, 5 µg/0.075 µg kg^−1^ min^−1^, *n* = 61) and High-Bolus Low-Infusion (Group HBLI, 10 µg/0.05 µg kg^−1^ min^−1^, *n* = 61)—according to different combinations of norepinephrine infusion and bolus doses. All patients received spinal anesthesia with 10 mg hyperbaric bupivacaine plus 12.5 µg fentanyl. Immediately after cerebrospinal fluid was obtained, patients underwent a norepinephrine protocol corresponding to the randomized group. The primary outcome was the incidence of post-spinal hypotension. Secondary outcomes were post-delivery hypotension, frequency of post-spinal hypertension and bradycardia, and neonatal outcomes. The incidence of post-spinal hypotension was 11.7% in Group HBLI, 6.7% in Group ZBHI and 1.7% in Group MBMI (*p* = 0.1). The overall incidence of post-delivery hypotension in parturients was 41.1% (*p* = 0.797). The lowest frequency of post-spinal bradycardia (8.3%) and hypertension (11.7%) was seen in Group HBLI. The neonatal APGAR scores at 1st minute were higher in Group MBMI than in Group ZBHI (8.58 vs. 8.23, *p* = 0.001). All three infusion and bolus dose combinations of norepinephrine effectively reduced the incidence of post-spinal hypotension. However, high-dose bolus (10 µg) followed by low-dose infusion (0.05 µg kg^−1^ min^−1^) of norepinephrine can be preferred due to the reduced frequency of bradycardia and hypertension during cesarean delivery under spinal anesthesia.

## 1. Introduction

The typical macrohemodynamic changes that occur in response to spinal anesthesia in parturients for caesarean section (C/S) include a rapid and profound decrease in systemic vascular resistance (SVR) and blood pressure, with a compensatory increase in cardiac output (CO) and heart rate (HR) [1]. Although intrathecal opioids were combined with local anesthetics to reduce these negative hemodynamic consequences of the high-quality spinal anesthesia, this did not solve the problem [2]. Considering these changes in the cardiovascular system, phenylephrine, which is used as a first-line agent in prevention and treatment of maternal hypotension, has become questionable as it causes a reflex decrease in HR and accordingly low CO state [3]. Norepinephrine (NE), however, has a weak β_1_-adrenergic agonistic effect in addition to its α_1_-adrenergic agonistic action, resulting in similar vasopressor efficacy to phenylephrine without negative chronotropic effects [4].

In recent years, the dose and method of administration of NE given in order to prevent maternal hypotension during C/S under spinal anesthesia have been the subject of extensive investigation. Because of its relatively short half-life of approximately 2.5 min, NE is generally given by continuous infusion in critical care settings [5]. However, as a result of administering vasopressors habitually as boluses in obstetric anesthesia, there is a trend for repeated bolus administrations of NE to prevent post-spinal hypotension (PSH) [6,7]. Conversely, a bolus dose followed by infusion of NE may be necessary to reach a steady-state plasma concentration and consequently to prevent PSH. Whether the dose or administration method of infusion only versus bolus followed by infusion of NE may influence the incidence of PSH without increasing side effects is not fully determined in the literature.

The primary objective of this study was to compare dosing protocol of NE administration, which included infusion only, low, or high bolus dose followed by low or high infusion dose combinations in terms of the incidence of PSH during C/S. The secondary aims of this study were to compare the residual effects of administering three different NE combinations on post-delivery hypotension (PDH), frequency of post-spinal bradycardia and hypertension, neonatal outcomes and number of interventions applied by the physician to overcome the hemodynamic deteriorations in each group.

## 2. Materials and Methods

This randomized, double-blinded, and dose–response trial was conducted between February 2021–May 2022 in Ankara University, School of Medicine, Department of Anaesthesiology, Ankara, Turkiye. Ethical approval (Ethical Committee 17-55-20) was obtained from Clinical Research Ethics Committee of Ankara University School of Medicine, Ankara; Turkiye (Chairperson Prof A. Ikinciogullari) on 9 September 2020, before commencing this study. The study protocol was registered at clinicaltrials.gov with the identification number NCT04701190. Finally, this study was approved by the Turkish Medicines and Medical Devices Agency.

**Figure 1:**
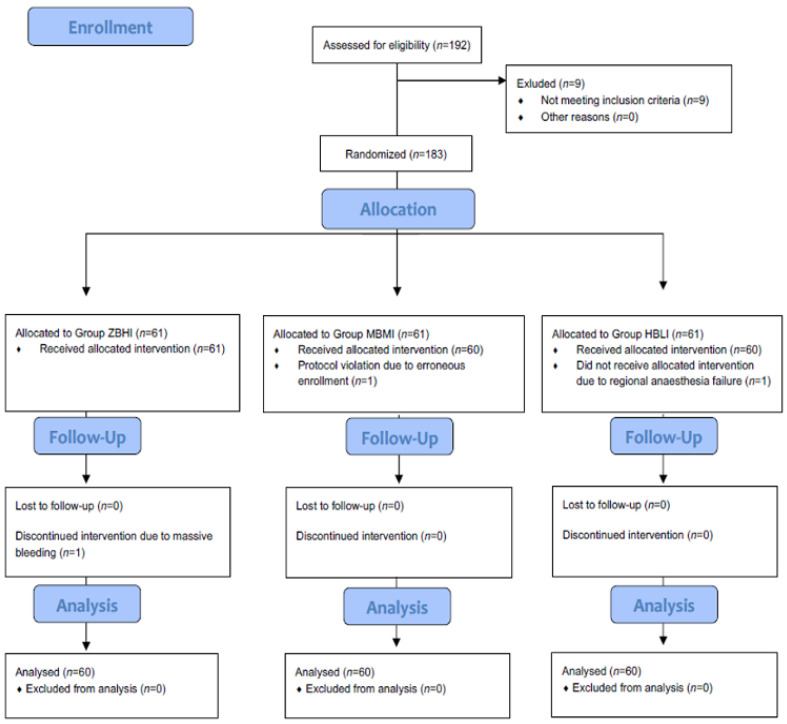
Consolidated Standards of Reporting Trials (CONSORT) chart showing patient recruitment and randomization. Group ZBHI–Group Zero Bolus High Infusion norepinephrine (NE) including 0 μg bolus dose plus 0.1 μg kg^−1^ min^−1^ infusion dose. Group HBLI—Group High Bolus Low Infusion NE including 10 μg bolus dose plus 0.05 μg kg^−1^ min^−1^ infusion dose. Group MBMI—Group Moderate Bolus Moderate Infusion NE including 5 μg bolus dose plus 0.075 μg kg^−1^ min^−1^ infusion dose.

Inclusion criteria were as follows: 18–40 years old and ASA-2 term pregnant women scheduled for elective C/S under spinal anesthesia. Parturient with ASA 3–6 class, any absolute contraindication to spinal block, neurological or cardiac disorder, basal systolic blood pressure above 140 mmHg or below 100 mmHg, peripartum bleeding or emergent situations both for fetus and mother, BMI > 40, allergy to study drugs, with a sensory block level lower than T6 at 20 min after spinal anesthesia and parturient who did not give consent were excluded from this study. After obtaining written informed consent, all demographics were recorded and the patients were randomly assigned to 3 groups, using the sealed opaque envelopes containing the bolus and infusion doses of NE. Accordingly, patients were randomized to the Zero-Bolus High-Infusion Group (Group ZBHI, *n* = 61) in which patients would receive 0.1 µg kg^−1^ min^−1^ infusion of NE without a bolus dose, the Moderate-Bolus Moderate-Infusion Group (Group MBMI, *n* = 61) in which patients would receive a 5 µg initial bolus dose followed by 0.075 µg kg^−1^ min^−1^ infusion of NE or the High-Bolus Low-Infusion Group (Group HBLI, *n* = 61) in which patients would receive a 10 µg initial bolus dose followed by 0.05 µg kg^−1^ min^−1^. The randomization scheme is shown in Figure 1.

After admission to the operating room, all patients were placed under standard American Society of Anaesthesiologist (ASA) monitoring including 3-lead electrocardiography, pulse oximetry and non-invasive blood pressure (Infinity C500; Drager, Lübeck, Germany). Baseline values of systolic blood pressure (SBP), diastolic blood pressure (DBP), mean blood pressure (MBP) and heart rate (HR) were measured in supine position with left uterine displacement and recorded as the arithmetic mean of three consecutive measurements at least 2 min intervals with a difference of less than 10%. As per standard practice, SBP, DBP, MBP, HR and pulse oximetry measurements were recorded in every 2 min throughout the surgery. After insertion of an 18-gauge cannula into a large vein outside the elbow area, balanced crystalloid solution was started to infuse rapidly. Subarachnoid block was performed through L3-4 or L4-5 intervertebral space in the sitting position. A solution of 10 mg 0.5% hyperbaric bupivacaine plus 12.5 mg fentanyl was injected into subarachnoid space using a 25-gauge pencil point spinal needle (PenPen, Egemen International, İzmir, Turkiye). After completion of subarachnoid injection, patients were immediately placed supine on the operating table with a wedge under right buttock. The interval between intrathecal injection and reaching sensory level of T4 was recorded.

To assure blinding, the study group was known only to the research assistant, who would be responsible for each step of the NE administration including starting dose of NE infusion, applying a bolus dose or cessation of the infusion of NE according to the study protocol. Initially, research assistant carefully diluted NE (Cardenor, 4 mg/4 mL; Vem İlaç, İstanbul, Turkiye) into 100 mL 5% dextrose and then set the necessary adjustments on the infuser (Infusomat^®^ Space, BBraun, Germany) according to the NE dose chart of the patient group to which was randomized. Another physician who was unaware of the patients’ group gave all the necessary information to the research assistant about the need for the bolus dose of NE or cessation of infusion of NE according to the data related to the changes in the parturients’ blood pressure.

The study drug was connected to a three-way stopcock that was directly attached to the intravenous catheter and started to be administered simultaneously with the detection of cerebrospinal fluid (CSF) free flow during subarachnoid block. NE infusion was stopped 5 min after delivery in all patients. Accordingly, patients randomized to Group ZBHI received an NE infusion of 0.1 µg kg^−1^ min^−1^ without a bolus dose. Patients randomized to Group MBMI received a 5 μg NE bolus followed by NE infusion of 0.075 µg kg^−1^ min^−1^. Patients randomized to Group HBLI received a 10 μg NE bolus followed by NE infusion of 0.05 µg kg^−1^ min^−1^.

The intraoperative period was divided into 2 consecutive periods. The first period was called the post-spinal period and included the period between the completion of subarachnoid block and the time of delivery. The subsequent period between the time of delivery and the end of the surgery was called the post-delivery period. All complications were entitled according to the period in which they occurred. Accordingly, PSH and PDH were defined as a reduction in SBP of 20% or more of the baseline value before and after the delivery, respectively. Similarly, post-spinal (PSSH) and post-delivery severe hypotension (PDSH) were defined as a decrease in SBP of 40% or more from the baseline value before and after the delivery, respectively. During these two periods, all interventions to treat the hemodynamic disturbances were applied according to the study protocol by the anesthesiologist who was blinded into the patient group. If PSH or PDH was developed, a bolus of 10 mg ephedrine was administered. If hypotension persisted after 2 min, a repeated dose of 10 mg ephedrine i.v. was administered again. If PSSH or PDSH occurred, 15 mg ephedrine i.v. bolus was administered. In case of bradycardia (HR < 60 beats min^−1^) without maternal hypotension, NE infusion was stopped. If bradycardia persisted for 2 min despite discontinuation of NE infusion, 0.5 mg of atropine i.v. bolus was administered. Moreover, if the heart rate fell below 50 beats min^−1^, 0.5 mg atropine i.v. bolus was administered without waiting for 2 min. As soon as the HR increased above 60 beats min^−1^, NE infusion was started again at the same infusion dose corresponding to the NE group that the patient randomized. If bradycardia was accompanied by hypotension, 10 mg ephedrine i.v. bolus was administered. In contrast to hypotension, if the SBP was found to be 20% above the basal value, the NE infusion was stopped and recorded as a hypertensive episode. If the systolic blood pressure fell below this determined hypertensive value, NE was restarted at the same dose. Hemodynamic changes mentioned above and the number of interventions including administration of ephedrine or atropine, stopping or re-starting of NE infusion, which applied by the physician to overcome the hemodynamic consequences of spinal anesthesia were all recorded until the patient left the operating room. After delivery of the newborn, 10 IU i.v. oxytocin infusion and 0.2 mg intramuscular methylergonovine were given. NE infusion was stopped 5 min after delivery in all patients.

### 2.1. Outcomes

The primary outcome of this study was to compare the effects of three different NE administration protocols on the incidence of PSH in pregnant women who were delivered by elective C/S, and to reveal the most effective NE protocol that reduced the incidence of PSH.

The secondary outcomes of this study were to compare the effects of three different NE administration protocols on the incidence of PDH, frequency of post-spinal bradycardia and hypertension, neonatal outcomes regarding the 1st and 5th minutes APGAR scores and the number of interventions applied by the physician for patients with hemodynamic deterioration.

### 2.2. Statistical Analysis

In this study, power analysis of the incidence of PSH between groups was performed using the chi-square test. Accordingly, 51 patients per group, with a power of 0.80, were included in this study. Despite the possibility that patients who were likely to be excluded from this study for various reasons constitute 20% of the entire study population. For this reason, a total of 183 patients, 61 patients in each study arm, were included in this study.

Statistical Package for Social Science software, version 15 for Microsoft Windows (SPSS Inc., Chicago, IL, USA), was used for data analysis. Categorical data were expressed as frequency (%). Continuous data were tested for normality using the Shapiro–Wilk test and presented as either mean (SD) or median (quartiles) as appropriate. The primary outcome (frequency of PSH) was analyzed using the chi-square test. Secondary outcomes (frequency of bradycardia, reactive hypertension, nausea, and vomiting) were analyzed using the chi-square test or Fisher’s exact test as deemed appropriate. Continuous data were analyzed using one-way ANOVA with post hoc Tukey modification (for normally distributed data) and using the Kruskal–Wallis test on ranks (for skewed data). For repeated measures, a two-way repeated-measures ANOVA was used to evaluate dose (between-groups factor) and time (repeated measures). Post hoc pairwise comparison was performed using Bonferroni test. A *p* value of less than or equal to 0.05 was considered statistically significant.

## 3. Results

One hundred and ninety-two parturients were enrolled into this study. Nine of them were excluded due to the exclusion criteria of the study protocol. Figure 1 shows the details of patient randomization scheme and flow chart of the present study. One patient in Group ZBHI due to massive bleeding caused by uterine atony and the other one in Group HBLI due to failed spinal anesthesia were excluded from this study. A research assistant applied erroneous NE bolus and infusion combination, one patient in Group MBMI was also excluded from this study. Finally, sixty patients were analyzed in each group. The demographic and operative data were comparable in each study groups (Table 1).

**Figure 2:**
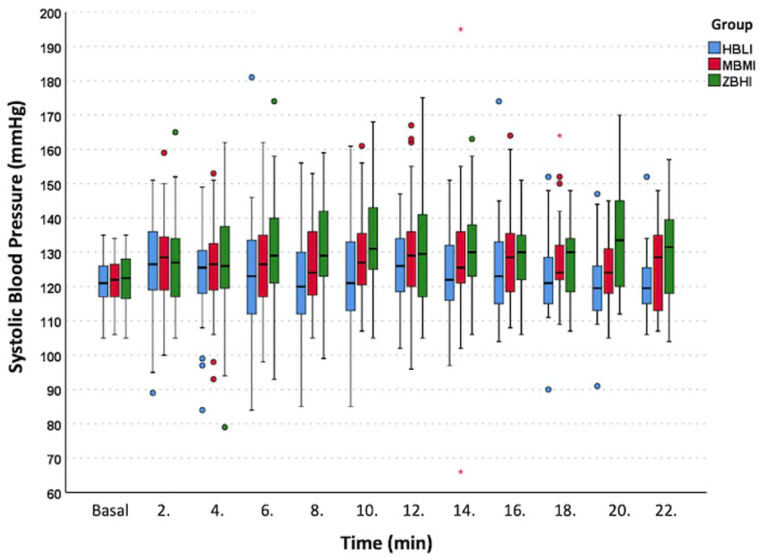
Systolic blood pressure trend. Group ZBHI—Group Zero Bolus High Infusion norepinephrine (NE) INCLUDION 0 μg bolus dose plus 0.1 μg kg^−1^ min^−1^ infusion dose. Group MBMI—Group Moderate Bolus Moderate Infusion NE including 5 μg bolus dose plus 0.075 μg kg^−1^ min^−1^ infusion dose. * represents high extreme SBP values in Group MBMI. Boxplot: line withing the box; median value, box top-bottom lines 25–75% top and bottom whiskers line; min-max value. Blue, red and green colored dots represent potential outliers.

In the post-spinal period, the overall incidence of PSH was 6.7% and there was no statistically significant difference between the three groups in terms of PSH frequency, the lowest frequency was 1.7% in Group MBMI, and the highest frequency was 11.7% in Group HBLI (*p* = 0.1). There was no patient developing PSSH in Group ZBHI and Group HBLI, with one patient (1.7%) in Group MBMI. The number of patients with hypertensive episodes during this period was statistically lower in Group HBLI (11.7%) compared to Group ZBHI (33.3%, *p* = 0.008) and Group MBMI (33.3%, *p* = 0.008). Hypertension was observed in all patients at least once and at most 5 times in Group MBMI. Figure 2 showed that the SBP trend was more stable and closer to the baseline in Group HBLI, compared to Group ZBHI and Group MBMI. Similar to hypertension, there was a tendency in the frequency of bradycardic episodes to be lower in Group HBLI (8.3%) compared to Group ZBHI (23.3%, *p* = 0.08) and Group MBMI (18.3%, *p* = 0.08) but there was no statistically significant difference between the groups. Among the bradycardic patients, the group with the lowest number of patients who needed atropine was in Group HBLI compared to Group ZBHI and Group MBMI (20% of 5 vs. 35.7% of 14 vs. 45.5% of 11 patients, respectively; *p* = 0.69).

In the post-delivery period, PDH was developed with an overall incidence of 41.1% in the study population. Although there was no significant difference between groups, the lowest incidence of PDH was 38.3% in the Group HBLI (*p* > 0.05). Moreover, PDSH was not found in any of the patients. In contrast to post-spinal period, the lowest incidence of bradycardia was recorded in Group MBMI (1.7%, *p* = 0.30) in this period. However, atropine need was highest in Group MBMI (100% of 1 patient). In the period from spinal induction to delivery, no significant difference was found in terms of nausea and vomiting in all three groups (15.0%, 16.7%, and 23.7%, respectively; *p* = 0.418). In both periods, the number of physician intervention was similar between the groups. The parameters of maternal outcomes recorded in post-spinal and post-delivery periods are summarized in (Table 2).

The mean of neonatal APGAR score (1 min) score was above 7 in all groups. In Group MBMI, there were two neonates with an APGAR score below 7. The group with the lowest APGAR score among the groups was Group ZBHI, and there was a statistically significant difference between the Group ZBHI and Group MBMI (*p* < 0.001). The neonatal APGAR (5 min) score was above 9 in all groups, and there was no significant difference between the groups (*p* > 0.05) (Table 3).

According to the secondary analysis of the data, in different scenarios where hypotension was defined according to SBP as being below 100 mmHg or MBP as being below 65 mmHg instead of a decrease in SBP for more than 20% according to the basal measurement, the number of patients to be considered hypotensive increased in all three groups, and the rate of occurrence of hypotension was the lowest in the Group MBMI (6.6%). The change in PSH incidence is shown in (Table 4) according to different hypotension definitions. Another analysis of the data revealed that the distribution of patients according to their body weight divided into 10 kilos and the incidence of PSH in these weight-based groups were not different between the three groups (Table 5).

## 4. Discussion

In this randomized, double-blind, graded dose–response study of NE, 10 µg bolus followed by 0.05 µg kg^−1^ dk^−1^ infusion as given to parturient simultaneously with spinal anesthesia was shown to be effectively reduced the incidence of maternal PSH with low risk of bradycardia, hypertension and atropine requirement compared to other two administration protocols of NE.

The current rate of cesarean delivery is 51.2% in Turkiye, which is approximately three times the consensus recommendation rate made by World Health Organization [8]. Considering that spinal anesthesia is the most frequently used technique for C/S delivery, this high C/S rate means increased number of parturient and neonates potentially harmed by the adverse effects of the spinal anesthesia itself. The most frequent adverse effect of spinal anesthesia is maternal hypotension with the incidence of varying between 7.4% and 74.1% in a cohort of women with elective cesarean section [9]. Since the early 2000s, a physiology-based treatment approach to PSH including the use of vasopressor and fluid co-loading has been initiated in order to prevent hypotension and its consequences on the mother (nausea, vomiting, and dizziness) and the neonate (fetal acidosis, fetal hypoxia) [10]. As stated in the current international consensus statement, vasopressors should be used routinely and preferably prophylactically to eliminate the risk of maternal hypotension during C/S under spinal anesthesia [11]. In this context, phenylephrine has been used for decades against the PSH. However, giving especially high-dose phenylephrine in order to keep blood pressure at baseline might be questioned due to its lowering effects on heart rate and consequently cardiac output [12]. Finally, the use of NE is very popular and the scientific evidence on this topic has been increasing rapidly in the last 10 years.

In fact, NE was first studied aiming not to prevent but to treat PSH effectively in 2006. However, this clinical study was published almost 10 years after it was studied [13]. Additionally, the first randomized double-blind study, in which the effects of NE and phenylephrine on maternal CO were compared, showed that NE increased CO by creating a median difference of 9.8% compared to phenylephrine, and this was mainly caused by increasing maternal HR [4]. Therefore, it can be speculated that NE should be the first-choice vasopressor in the prevention of PSH, due to its similar effects on blood pressure maintenance with a reduction in the undesirable negative chronotropic effects of phenylephrine. Unfortunately, this certainty observed in the drug choice in favor of NE does not exist regarding the dose of NE and whether it should be given as a bolus or infusion only, or bolus followed by infusion doses to prevent maternal PSH.

Considering that the negative hemodynamic consequences of spinal anesthesia might last up to approximately 30 min, NE should be given as a continuous infusion with or without a bolus dose [14]. The rationale behind the continuous mode of NE administration instead of intermittent bolus doses is that 2.5 min of a short half-life of NE, and therefore a continuous infusion mode, is desirable to maintain a constant plasma level [5]. In a recent dose-finding study, three different infusion doses of NE were administered to prevent PSH in 284 parturient. All patients were randomized into 0.025, 0.05 and 0.075 µg kg^−1^ min^−1^ NE following a 5 µg standard bolus dose of NE. The lowest frequency of PSH was 24.7% when NE was given as 0.05 µg kg^−1^ min^−1^ following a 5 µg bolus at the same time as spinal injection of hyperbaric bupivacaine [15]. Although NE is applied preventively against hypotension, the PSH still occurs in one out of every four pregnant women, which seems a high incidence. These data inspired the research question of our study. We first hypothesized that increasing the bolus dose of NE would decrease the incidence of PSH without any further increase in side effects of NE. Warwick D. Ngan Kee [16] calculated ED50 and ED90 values for the NE bolus dose to treat PSH as 10 µg and 18 µg, respectively. Therefore, we decided to investigate the PSH incidence while administering 0.05 µg kg^−1^ min^−1^ infusion following 10 µg bolus instead of 5 µg as in Hasanin et al. trial [15]. This simple and feasible approach has halved the incidence of PSH from 24.7% to 11.7% in Group HBLI of our study. However, the bradycardic episodes were more frequent in our study compared to Hasanin AM et al. [15] (8.3% vs. 3.2%). This difference in bradycardia incidence might be attributable to different definitions of bradycardia in these two studies. The bradycardia was defined as the HR below 60 min^−1^ in our study, whereas it was accepted as bradycardia if the HR fell below 55 min^−1^ in Hasanin AM et al. study [15].

Sundararajan M. et al. [17] used infusion method only without an initial bolus dose of NE to prevent the PSH during C/S. They used 0.05 µg kg^−1^ min^−1^ of NE starting at the same time as spinal injection of local anesthetic. The incidence of PSH was lower in NE-infused group compared to the control group (11.1% vs. 33.3%). As the authors used 9 mg 0.5% hyperbaric bupivacaine without opioid for spinal anesthesia, which was lower than the dose used in our study, they might have founded a similar incidence of PSH as in the Group HBLI in our study (11.1% vs. 11.7%). The 10 µg extra initial bolus NE in Group HBLI might have prevented further blood pressure reduction due to the use of 10 mg instead of only 9 mg hyperbaric bupivacaine for spinal anesthesia. Furthermore, Chen Y. et al. [18] administered an extra 6 µg initial bolus dose followed by 0.05 µg kg^−1^ min^−1^ of NE starting at the same time as 0.5% 12.5 mg bupivacaine and found a higher PSH incidence of 17.53% compared to our result. Thus, it might be speculated that as the local anesthetic dose is increased, the need for a vasopressor drug might be increased; in particular, an initial bolus dose might be used to stabilize the maternal blood pressure.

In our study, we revealed that the incidence of PSH was decreased from 11.7% to 6.7% when NE was given as an infusion of 0.1 µg kg^−1^ min^−1^ without a bolus dose in Group ZBHI compared to Group HBLI. However, the highest hypertension and bradycardia frequencies were occurred in Group ZBHI compared to Group HBLI (33.3% vs. 11.7%; 23.3% vs. 8.3%) and Group MBMI (33.3% vs. 33.3%; 23.3% vs. 18.3%). Doubling the infusion dose of 0.05 µg kg^−1^ min^−1^ to 0.1 µg kg^−1^ min^−1^ without an initial bolus dose of NE might be decreased the incidence of PSH [17]. Fu F. et al. [19] found a similar incidence of PSH in patients receiving infusion 0.08 µg kg^−1^ min^−1^ without a bolus dose of NE to the incidence that we found in Group ZBHI. Similar to the incidence of PSH, the rate of reactive hypertension was also found to be similar in the mentioned groups of both studies. As a result, due to the side effects of NE itself, it may be recommended not to use the high-dose NE infusion, which was used in both our and the Fu F et al.’s studies [19] unless it is necessary.

The present study showed that PSSH could be avoided by all three NE administration protocols. Hence, in Group ZBHI and HBLI no patients and in Group MBMI only one patient had developed PSSH. In our protocol, the NE was stopped 5 min after delivery in all patients and the incidence of PDH was 40%, 38.3% and 45% in Group ZBHI, Group HBLI and Group MBMI, respectively. As NE has a short half-life, a high percentage of parturients required NE retreatment after delivery. Although the NE cessation protocol was similar to the two trials performed by Hasanin AM et al. [15,20], their PDH incidence was lower than the overall PDH incidence of our study. This may have occurred mainly due to the different uterotonics and fluid amount given to the patients extending beyond the delivery. Therefore, it is reasonable to apply liberal fluid treatment especially in patients with longer fasting times, combined with an extended duration of NE infusion beyond the delivery to prevent PDH. In addition, rapid breakdown following possibly higher plasma levels of NE compared to Hasanin’s studies might have resulted in rebound hypotension due to the effects of sympathectomy of spinal anesthesia that has not expired yet.

A prospective randomized, double-blind study detected the maximum decrease in SBP of the parturients occurred 5 to 6 min after starting the NE infusion of 0.05 µg kg^−1^ min^−1^ [17]. This might imply that a stable concentration of NE in the plasma is achieved by 5 to 6 min. Therefore, the authors recommended a bolus dose prior starting infusion of NE to prevent the initial hypotensive episodes due to spinal anesthesia. Interestingly, similar to the predictions of the authors, we can also conclude that administering high dose of bolus with simultaneous infusion of NE might decrease the number of PSH episode, the number of hypertensive episode and as a result the number of physician intervention. When the results of these two studies were evaluated together, it was thought that the combination of high-dose bolus and low-dose NE infusion could demonstrate maximum effect with low side-effect risk profile to prevent PSH.

One of the major consequences of hypotension is maternal discomfort due to nausea and vomiting. It was shown that vasopressor premedication could decrease the incidence of these side effects of spinal anesthesia [21]. In our study, the overall incidence was 18.4% and 3.9% for nausea and vomiting, respectively, without any significant difference between groups. These results are comparable with the results of Xu et al.’s study [22]. However, in another study, the authors found the incidence of nausea and vomiting of 9.4% in their patient population who had a 10 µg rescue bolus dose combined with 0.05 µg kg^−1^ min^−1^ NE with 10 mg metoclopramide premedication before spinal anesthesia [23]. In our patients given the same bolus and infusion dose of NE who were randomized into Group HBLI, the nausea incidence was found nearly twice the rate of the previous study as 16.7%. This discrepancy might be explained by the lack of premedication for PONV in our study.

The NE is not thought to readily cross the placenta into the fetus, because of the ability of the placenta to break down catecholamines [24]. However, the safety profile of NE for the fetus and neonate is still a potential concern. In real life, the Apgar scores at 1st and 5th minutes after birth are considered as a good indicator of wellbeing and safety of the neonate. There were 1 and 2 neonates had an APGAR score below 7 at 1st min in Group ZBHI and Group MBMI, respectively. The APGAR score at 1st min was lower in Group ZBHI compared to Group MBMI with a significance. However, this difference in APGAR score had disappeared at 5th minute. The possible explanation for the lower score at 1 min in Group ZBHI was the higher incidence of bradycardia compared to Group MBMI. However, the literature and our clinical experience demonstrate that the 5th minute APGAR score is more important and reliable to anticipate the relative risk of cerebral palsy [25].

A total of 15 different definitions of hypotension which occurred after spinal anesthesia in parturients were used in the literature. A decrease in systolic arterial pressure below 80% of the baseline value and a systolic blood pressure below 100 mmHg were the most frequently used definitions [9]. In our patient population, a secondary analysis of hemodynamic data according to different definitions of hypotension revealed that none of the three definitions differ in detecting hypotension. However, in patients given a high bolus dose of NE, 4 more patients, even they were under NE treatment protocol, were considered to develop hypotension according to definition of SBP below 100 mmHg. However, the MBP seemed to be preserved probably due to a 10 µg bolus dose of NE in these patients. Klöhr et al. [9] found a 11.1% difference in PSH incidence between these two definitions in their patient population with serial measurements performed at 1 min intervals after spinal anesthesia. In the present study, this difference has almost halved the incidence of Klöhr’s study from 11.1% to 6.6%. The main reason for this discrepancy would be the presence of very well protocolized vasopressor treatment in our study compared to the treatment left to the discretion of the attending anesthesiologist. Additionally, the low PSH incidence in our study might be the result of measurement intervals which were larger than the abovementioned study. Therefore, it is possible to underestimate the decreases in SBP measurements.

The effect of NE on blood pressure was initially thought to be well correlated with its dosing, where a higher dose resulted in higher blood pressure [26]. Due to its low volume of distribution and affecting only the sympathetic nervous system tissues, it can be theoretically assumed that the body weight does not play a crucial role in the occurrence of the effects of NE and therefore can be ignored in its dosing. In 2016, American Society of Health-System Pharmacists standardized infusion concentrations of various medications including NE infusion. They recommended weight-based dosing of NE. However, there is no consensus about which NE dosing strategy should be standard of care. In a retrospective cohort study, 189 critically ill patients’ data related to NE dosing according to weight based or non-weight-based dosing were studied. The authors found that a lower NE dose was used to achieve the blood pressure target in weight-based dosing of NE [27]. Accordingly, we evaluated whether the body weight of the parturient affects the incidence of PSH due to the alterations in the dosing of NE, which has a lower volume of distribution. However, there is no difference in terms of PSH incidence between three groups according to the analysis of their actual body weights. Hence, weight-based dosing of NE in parturient to prevent PSH could be used confidently. However, whether weight-based and non-weight-based dosing are superior to each other in this special patient population is not clear and this topic should be studied in the future.

This single-center study has several limitations. First, the hemodynamic changes due to spinal anesthesia and subsequently given NE administration were followed as major changes in SBP and HR of the parturient. However, the most important parameter that should be determined and examined, might be CO or stroke volume during post-spinal period. Moreover, although NE was given in different bolus and infusion doses to all patients, there were still an important number of patients experiencing nausea and vomiting. Although risk scoring systems for PONV are generally used in patients undergoing general anesthesia, the risk factors according to the multivariate analysis of patients undergoing spinal anesthesia were found as high baseline HR and being a non-smoker [28]. Even if all pregnant women are considered as non-smokers, it should be considered that this may not be true for all. Therefore, recording and analyzing smoking status and other risk factors related to PONV in parturients might be recommended for future studies. Due to the strict regulations related to ethics approval for the child population in the country, the newborns were evaluated with a clinical score instead of umbilical arterial sample analysis. However, it would be more accurate to determine the umbilical artery pH of the newborns to make precise comments on this issue. Lastly, the PDH is still a problem in this special patient population. Although the patients were considered as healthy due to their younger ages and careful medical attention for 9 months, perioperative hypotension might be the triggering event for postoperative major complications. However, the consequences of hypotension occurred in this period except nausea and vomiting were not determined in the present study. This could be considered as the most unreliable point of this study and should be examined as a safety measure for mothers in future studies.

## 5. Conclusions

This study has demonstrated that a 10 µg bolus followed by 0.05 µg kg^−1^ dk^−1^ infusion of NE effectively prevents hypotension in patients undergoing C/S following spinal anesthesia performed with 10 mg 0.5% hyperbaric bupivacaine plus 12.5 mg fentanyl, with a low risk of adverse effects of NE such as hypertension and bradycardia.

## Figures and Tables

**Table 1:** Demographic and operative data.

	Group ZBHI (*n* = 60)	Group MBMI (*n* = 60)	Group HBLI (*n* = 60)	*p*
Age, yr	30.18 ± 5.23 [30 (20–40)]	31.02 ± 4.49 [32 (21–39)]	30.27 ± 5.17 [30 (20–39)]	0.511
Height, cm	162.5 ± 6.55 [163.5 (140–176)]	161.7 ± 4.81 [160.0 (153–174)]	162.8 ± 5.45 [162.5 (153–176)]	0.439
Weight, kg	78.47 ± 10.92 [78.5 (53–100)]	76.52 ± 11.59 [78 (52–104)]	78.13 ± 10.99 [76 (55–110)]	0.494
IT injection to sensory level of T4 interval (min)	6.12 ± 2.08 [6 (2–11)]	6.63 ± 2.93 [6 (2–17)]	6.32 ± 2 [6 (2–12)]	0.737
Incision to delivery interval (min)	5.45 ± 2.75 [5 (2–13)]	4.67 ± 2.51 [4 (2–15)]	5.63 ± 2.83 [5 (2–13)]	0.088
Surgery duration (min)	30.38 ± 8.52 [29.5 (16–58)]	28.25 ± 9.58 [26 (14–65)]	30.47 ± 8.58 [28.5 (16–60)]	0.085
Baseline heart rate (beats/min)	93.5 (70–130)	97 (72–125)	92 (68–126)	0.624
Baseline SBP (mmHg)	122.5 (105–135)	122 (106–134)	121 (105–135)	0.844

Data are the mean ± SD and the median (range); IT, intratechal; SBP; systolic blood pressure. Group ZBHI, Group Zero-Bolus High-Infusion norepinephrine (NE) including 0 µg dose plus 0.1 µg kg^−1^ min^−1^ infusion dose; Group HBLI, Group High-Bolus Low-Infusion NE including 10 µg dose plus 0.05 µg kg^−1^ min^−1^ infusion dose; Group MBMI, Group Moderate-Bolus Moderate-Infusion NE including 5 µg dose plus 0.075 µg kg^−1^ min^−1^ infusion dose.

**Table 2:** Maternal outcomes.

	Hemodynamic Change	Group ZBHI *n* = 60	Group MBMI *n* = 60	Group HBLI *n* = 60	Total *n* = 180	*p*
Post-spinal period	PSH, *n* (%)	4 (6.7)	1 (1.7)	7 (11.7)	12 (6.7)	0.100
	Number of PSH episode, *n*	1.25 ± 0.50	1.43 ± 0.79	1.00 ± 0.0	1.33 ± 0.65	0.825
	PSSH, *n* (%)	0 (0)	1 (1.7)	0 (0)	1 (1.7)	1.000
	Hypertension, *n* (%)	20 (33.3)	20 (33.3)	7 (11.7)	47 (26.1)	0.008
	Number of hypertensive episode, *n*	1.47 ± 0.70	1.70 ± 1.17	1.00 ± 0.0	1.50 ± 0.9	0.180
	Bradycardia, *n* (%)	14 (23.3)	11 (18.3)	5 (8.3)	30 (16.7)	0.080
	Number of bradycardic episode, *n*	1.21 ± 0.58	1.25 ± 0.75	1.20 ± 0.45	1.23 ± 0.62	0.976
	Atropine requirement, *n* (%)	5 (35.7)	5 (45.5)	1 (20.0)	11 (36.7)	0.698
	Physician intervention, *n*	0.93 ± 1.26	0.75 ± 1.55	0.60 ± 1.09	0.76 ± 1.31	0.114
Post-delivery period	PDH, *n* (%)	24 (40)	27 (45)	23 (38.3)	74 (41.1)	0.797
	PDSH, *n* (%)	0 (0)	0 (0)	0 (0)	0 (0)	1.000
	Bradycardia, *n* (%)	5 (8.3)	1 (1.7)	3 (5)	9 (5)	0.302
	Number of bradycardic episode, *n*	1.20 ± 0.45	1.00 ± 0.0	1.33 ± 0.58	1.22 ± 0.44	0.796
	Atropine requirement, *n* (%)	2 (40.0)	1 (100)	2 (66.6)	5 (55.5)	1.000
	Physician intervention, *n*	0.83 ± 1.12	1.00 ± 1.20	1.18 ± 1.37	1.01 ± 1.23	0.445
	Nausea, *n* (%)	9 (15)	14 (23.7)	10 (16.7)	33 (18.4)	0.418
	Vomiting, *n* (%)	4 (6.7)	1 (1.7)	2 (3.3)	7 (3.9)	0.507

Group ZBHI, Group Zero-Bolus High-Infusion norepinephrine (NE) including 0 µg dose plus 0.1 µg kg^−1^ min^−1^ infusion dose; Group HBLI, Group High-Bolus Low-Infusion NE including 10 µg dose plus 0.05 µg kg^−1^ min^−1^ infusion dose; Group MBMI, Group Moderate-Bolus Moderate-Infusion NE including 5 µg dose plus 0.075 µg kg^−1^ min^−1^ infusion dose. PSH, post-spinal hypotension; PSSH, post-spinal severe hypotension; PDH, post-delivery hypotension; PDSH, post-delivery severe hypotension; NE, norepinephrine.

**Table 3:** Neonatal outcomes.

	Group ZBHI *n* = 60	Group MBMI *n* = 60	Group HBLI *n* = 60	Total *n* = 180	*p*
APGAR score at 1 min *	8.23 ± 0.53 # 8.00 (7–10)	8.58 ± 0.50 9.00 (8–9)	8.45 ± 0.53 8.00 (7–9)	8.42 ± 0.54 8.00 (7–10)	0.001
APGAR score < 7 at 1 min	1 (1.7)	2 (3.3)	0 (0)	3 (1.7)	
APGAR score at 5 min *	9.40 ± 0.56 9.00 (8–10)	9.52 ± 0.50 10.00 (9–10)	9.63 ± 0.52 10.00 (8–10)	9.52 ± 0.53 10.00 (8–10)	0.055

Group ZBHI, Group Zero-Bolus High-Infusion norepinephrine (NE) including 0 µg dose plus 0.1 µg kg^−1^ min^−1^ infusion dose; Group HBLI, Group High-Bolus Low-Infusion NE including 10 µg dose plus 0.05 µg kg^−1^ min^−1^ infusion dose; Group MBMI, Group Moderate-Bolus Moderate-Infusion NE including 5 µg dose plus 0.075 µg kg^−1^ min^−1^ infusion dose. #: Significantly differences between Group ZBHI and Group MBMI, (*p* < 0.05). For the variables with *, descriptive statistics were given as the mean ± SD [median (min-max)], otherwise frequency (percent).

**Table 4:** The PSH incidences according to different definitions of hypotension.

	Group ZBHI *n* = 60	Group MBMI *n* = 60	Group HBLI *n* = 60	*p*
Definition 1, *n* (%)	4 (6.7)	1 (1.7)	7 (11.7)	0.100
Definition 2, *n* (%)	5 (8.3)	3 (5)	11 (18.3)	0.047
Definition 3, *n* (%)	5 (8.3)	4 (6.7)	7 (11.7)	0.619

Definition 1: Decrease in systolic blood pressure more than 20% according to the baseline. Definition 2: Systolic blood pressure below 100 mmHg. Definition 3: Mean blood pressure below 65 mmHg. Group ZBHI, Group Zero-Bolus High-Infusion norepinephrine (NE) including 0 µg dose plus 0.1 µg kg^−1^ min^−1^ infusion dose; Group HBLI, Group High-Bolus Low-Infusion NE including 10 µg dose plus 0.05 µg kg^−1^ min^−1^ infusion dose; Group MBMI, Group Moderate-Bolus Moderate-Infusion NE including 5 µg dose plus 0.075 µg kg^−1^ min^−1^ infusion dose.

**Table 5:** The PSH incidences according to the actual body weights.

	Body Weights (kg)					
	50–59	60–69	70–79	80–89	90–99	≥100
Group ZBHI	2 (0%)	10 (10%)	19 (10.5%)	16 (6.2%)	12 (0%)	1 (0%)
Group HBLI	1 (0%)	11 (27.3%)	22 (9.1%)	19 (5.3%)	5 (20%)	2 (0%)
Group MBMI	1 (0%)	16 (0%)	16 (6.2%)	19 (0%)	6 (0%)	2 (0%)

Data are given as the total number of patients in subgroup of patients according to their weight and the percentage of the patients who developed PSH (%) group of patients according to their weight.

## Data Availability

The datasets generated during and/or analyzed during the current study are available from the corresponding author on reasonable request.
